# Better understanding determinants of dietary guideline adherence among Dutch adults with varying socio-economic backgrounds through a mixed-methods exploration

**DOI:** 10.1017/S1368980023000228

**Published:** 2023-06

**Authors:** Josine M Stuber, Jeroen Lakerveld, Joline WJ Beulens, Joreintje D Mackenbach

**Affiliations:** 1 Amsterdam UMC location Vrije Universiteit Amsterdam, Epidemiology and Data Science, De Boelelaan 1117, Amsterdam, The Netherlands; 2 Amsterdam Public Health, Amsterdam, The Netherlands; 3 Julius Center for Health Sciences and Primary Care, University Medical Center Utrecht, Utrecht University, Utrecht, The Netherlands

**Keywords:** Dietary pattern, Prevention, Socio-economic status, Qualitative research

## Abstract

**Objective:**

Low dietary guideline adherence is persistent, but there is limited understanding of how individuals with varying socio-economic backgrounds reach a certain dietary intake. We investigated how quantitative and qualitative data on dietary guidelines adherence correspond and complement each other, to what extent determinants of guideline adherence in quantitative data reflect findings on determinants derived from qualitative data and which of these determinants emerged as interdependent in the qualitative data.

**Design:**

This mixed-methods study used quantitative questionnaire data (*n* 1492) and qualitative data collected via semi-structured telephone interviews (*n* 24). Quantitative data on determinants and their association with total guideline adherence (scored 0–150) were assessed through linear regression. Directed content analysis was used for qualitative data.

**Setting:**

Dutch urban areas.

**Participants:**

Adults aged 18–65 years.

**Results:**

A range of determinants emerged from both data sources, for example higher levels of cognitive restraint (*β* 5·6, 95 % CI 4·2, 7·1), habit strength of vegetables (*β* 4·0, 95 % CI 3·3, 4·7) and cooking skills (*β* 4·7, 95 % CI 3·5, 5·9), were associated with higher adherence. Qualitative data additionally suggested the influence of food prices, strong dietary habits and the social aspect of eating, and for the determinants cognitive restraint, habit strength related to vegetables, food prices and home cooking, some variation between interviewees with varying socio-economic backgrounds emerged in how these determinants affected guideline adherence.

**Conclusions:**

This mixed-methods exploration provides a richer understanding of why adults with varying socio-economic backgrounds do or do not adhere to dietary guidelines. Results can guide future interventions promoting healthy diets across populations.

Unhealthy diets are an established risk factor for chronic diseases^(1,2)^. Many countries have therefore implemented national dietary guidelines which aim to reduce chronic disease risk^(3)^. Yet, globally, dietary guideline adherence among adults is generally low^(4)^. To address low dietary guideline adherence, it is important to understand how individuals reach their current intake through an assessment of their broader dietary behaviours, such as food shopping, food purchasing and food preparation. For example, an observational study from the UK showed that home-prepared food consumption was associated with higher dietary quality^(5)^. Dietary behaviours are, in turn, influenced by behavioural determinants such as skills, budget and (taste) preferences, and more upstream determinants such as supermarket accessibility and social norms^(6,7)^. There is likely much variation in the ways in which and when individuals eat certain foods (e.g. through habits and meal planning^(8)^), if and how they try to eat healthy (e.g. based on nutrition knowledge^(9)^) or the perceived barriers in their daily eating routines (e.g. lack of money and skills^(10)^).

While structural factors such as financial, work and living circumstances should be acknowledged, it is known that some individual-level dietary determinants are unevenly distributed within populations, and there is some evidence for socio-economic differences in dietary behaviours^(11)^. For instance, observational data from France show that individuals with lower education levels spend more time on food preparation on a daily basis and that the lowest income group have less cooking equipment available than those with the highest income^(12)^. In a qualitative exploration, Australian males with lower education levels indicated to have limited cooking skills and nutrition knowledge and that their food intake was their autonomous choice (i.e. not affected by external messages or stimuli)^(13)^. On the other hand, those with a lower socio-economic position (SEP) may be more creative in working their way around a lack of certain resources. For example, a Dutch observational study showed that ethnic minority groups could achieve higher dietary guideline adherence at a lower cost than the Dutch ethnic majority group^(14)^. These observations point towards a need to better understand the interdependencies of different dietary determinants. Yet, there is generally very little insight into potential socio-economic variation in the importance and the variety of different dietary behaviours such as food shopping strategies (e.g. using a grocery shopping list) or food preparation activities (e.g. preparing meals at home) for dietary guideline adherence across populations. The current limited understanding of the complexity of how individuals with varying socio-economic backgrounds reach a certain intake hinders the translation of research insights and optimally tailoring of dietary interventional strategies, which could further increase the socio-economic inequalities in dietary intake^(15)^.

Systematically combining self-reported quantitative measures with findings from qualitative assessment on dietary behaviours can integrate, enrich and triangulate both types of results and provide more in-depth contextual understandings and multi-level perspectives within a real-life context^(16)^. However, mixed-methods studies on this topic are not available. Although the *Eet & Leef* study was not set up as a mixed-methods study^(11,17)^, its complementary quantitative and qualitative data provided us with a unique opportunity to investigate to what extent quantitative measurements of dietary guideline adherence and its determinants reflect the perceptions of individuals with varying socio-economic backgrounds thereof. We addressed the following objectives: (1) to identify how quantitative and qualitative data on adherence to dietary guidelines correspond and complement each other; (2) to explore to what extent determinants of dietary guideline adherence derived from quantitative data reflect findings on determinants derived from qualitative data and (3) to investigate the interdependence of determinants as identified in qualitative data.

## Methods

### Study design

This mixed-methods study was conducted according to a convergent parallel design^(18)^. We used data of the cross-sectional *Eet & Leef* study^(11,17)^, in which quantitative and qualitative data were collected independently from the same study sample during the same phase of the study process. The *Eet & Leef* study was part of the ‘Healthy Food Environments’ project funded by the Netherlands Organisation for Scientific Research (NWO Veni grant received by JDM). The overall aim of this 3-year project was to study several aspects of food choices, the food environment and health outcomes. For the current study, results from both the quantitative and qualitative data were analysed independently and compared following an integrated analytical approach described further down, to determine to what extent quantitative measurements of dietary guideline adherence and its determinants reflect the perceptions of individuals with varying socio-economic backgrounds thereof. The data comparison and interpretation of findings followed an iterative process, in which we compared qualitative and quantitative findings in a reflexive manner without pre-established criteria for corresponding, complementary or contradictory findings^(19)^. Reporting of this paper is in accordance with the *Mixed Methods Article Reporting Standards* published by the American Psychological Association^(20)^.

### Study population

The data were collected in the fall of 2019 based on a cross-sectional survey among an adult general population living in urban areas in the Netherlands (*n* 1492). An inclusion criterion was being aged 18–65 years. Exclusion criteria were not having the ability to understand the Dutch language and not having access to a computer with Internet and an e-mail address.

### Participant recruitment

A stepwise recruitment approach was applied. First, postal invitations were sent to ∼21 500 randomly selected home addresses in the twenty largest Dutch cities in terms of total population. Based on the socio-demographic characteristics of the respondents, males and lower-educated females appeared underrepresented. Therefore, as a second recruitment step, a social media campaign (Facebook and Instagram) was launched targeting lower and higher-educated males and lower-educated females. Third, fifty-four lower-educated males who participated in previous studies conducted at the same research department received a personal e-mail invitation to participate in the current study. Ultimately, 2533 individuals registered for participation out of which 2434 (96 %) appeared eligible for participation. Of those eligible, 1492 (61·3 %) participants completed all three parts of the survey which was deemed the analytical sample of the *Eet & Leef* study.

### Data collection

#### Quantitative data collection

Potentially eligible participants were directed to the project website. There they received the study information letter, provided informed consent for survey participation and could express their interest in participation in follow-up studies. Next, participants were asked for their current age and their e-mail address. Eligible participants received login details via e-mail for three web-based questionnaires about various dietary behaviours. Reminders were sent after 1 and 2 weeks in case of non-response. Upon registration, participants were informed they would receive a 7·50€ gift voucher of an online department store after completing all three parts of the survey.

For the current study, variables on socio-demographic characteristics of the study population included age (years), sex (male, female), occupational status (low-, medium- and high-level skill based on the ISCO08^(21)^), educational attainment (low: general secondary education, lower vocational education, or lower with < 12 years of education, medium: secondary vocational education and higher general secondary education with 12–16 years of education and high: higher professional education and university with > 16 years of education) and net household income (< 1200€, 1200–1800€, > 1800–2600€, > 2600–4000€, > 4000€). Net household income was adjusted for household members based on the OECD-modified equivalence scale^(22)^. Next, household member-adjusted income was categorised into low (≤ 1300€), medium (> 1300–2600€) and high (> 2600€).

SEP classification was based on the indicators income, education and occupation. As each of these indicators is considered to reflect a different underlying social component^(23)^, classification based on a summary score was considered the most suitable approach for the current study in order to capture the variety of these components. The summary score was created by the sum of the indicators income, education and occupation, which were all coded as ‘1’ for the low categories within the SEP indicators, ‘2’ for medium and ‘3’ for high (online Supplementary Table 1). Consequently, the summary score ranged from three to nine. Participants were classified into the categories low SEP (summary scores 3–5), medium SEP (6–7) and high SEP (8–9). All participants provided data for at least one SEP indicator. However, for those who missed data on one indicator (*n* 200), a low SEP was defined as a summary score of 2–3, medium SEP as 4 and high SEP as 5–6. For participants missing data on two indicators (*n* 15), classification was based on the single available indicator.

Adherence to the Dutch dietary guidelines was assessed using a validated thirty-four-item short FFQ – the Dutch Healthy Diet FFQ^(24)^ – measuring index scores of fifteen individual food group components. Each component is scored from zero (reflecting no guideline adherence for that specific component) to ten (reflecting optimal adherence). Their sum score (0–150) reflects total dietary guideline adherence via the Dutch Healthy Diet 2015-index score (DHD15-index score). The following determinants of dietary guideline adherence were included, for which summary scores were calculated: eating habits relating to uncontrolled eating (i.e. experienced difficulties with regulation of intake), emotional eating (i.e. overeating when feeling down) and cognitive restraint of eating (i.e. restrict intake to manage body weight), which were all measured with the validated three-factor eating questionnaire^(25)^. Other determinants were habit strength related to vegetable intake (validated self-report index of habit strength^(26)^), parental upbringing regarding dietary habits (items relating to household eating rules), cooking skills (validated self-perceived food literacy scale^(27)^), frequency of home cooking (never-always), frequency of not home cooking (i.e. ordering meals, eating out of home and preparing ready-to-eat meals), frequency of consuming of different types of snacks, shopping style (making shopping lists, weekly groceries at once, non-impulsive shopping) and taste preferences (sour, salt, sweet). Details on all questionnaire items, validity statistics and an interpretation of summary scores are presented in Supplementary Table 2.

#### Qualitative data collection

Upon completion of the survey, a selection of participants who expressed their interest in a follow-up study (*n* 1299) was iteratively invited for participation in qualitative interviews. Participants were divided into eight categories on the basis of sex (male, female), age (18–40 years, 41–65 years) and educational attainment (lower, higher), and we initially invited seven respondents per category to participate. Data of the first set of interviewees were collected and analysed after which it was decided that data saturation had not yet been reached. Non-responders or those hesitant to participate were politely reminded, but additional survey respondents were also invited. Ultimately, 135 participants were invited out of which twenty-four agreed to participate. After the data collection and analysis of these twenty-four interviews, JDM decided that data saturation was reached. Among the interviewees, a gift voucher of 15€ was allotted.

The qualitative data collection was based on semi-structured telephone interviews. All interviews were conducted between November 2019 and February 2020. Sixteen of the interviews were conducted by JDM and eight by a research assistant. JDM is a senior researcher in the research field of (nutritional) epidemiology and is trained and experienced in qualitative research methods. She provided informal training to the research assistant with a master’s degree in Public Health. Interviews were held until saturation of new information was reached. All interviews were scheduled at a moment convenient for the interviewee. At the start of the interview, the study aim was explained, and informed consent for study participation including audio recording was requested orally. Each interview lasted between 20 and 60 min, and recordings were transcribed verbatim. No member check (i.e. respondent validation of findings) was performed.

A semi-structured interview guide was used to identify dietary habits, changes over time and influences on those changes (online Supplementary Table 3). The interview started with opening and introductory questions which allowed interviewees to get acquainted with the questioning. Then, transition questions were used to guide interviewees towards discussing factors influencing their dietary habits. Questions were asked on (1) interviewees’ own description of their dietary habits (e.g. typical week and exceptions), (2) variations in dietary habits (e.g. weekdays *v*. weekend days, with *v*. without company), (3) individual characteristics related to dietary habits (e.g. shopping style, giving in to temptations) and (4) role of food in life (e.g. does it give pleasure, link with health). Researchers followed the interview guide during the interview but asked follow-up questions to obtain more in-depth information about the topics. Open-ended questions were used to leave space for additional themes to emerge.

### Data analyses

#### Quantitative data analysis

Descriptive statistics were generated for sex, age SEP and all determinants of dietary guideline adherence variables. Categorical variables were presented by their number and proportions, normally distributed continuous variables by their mean and standard deviation and non-normally distributed variables by their median and interquartile range. Cross-sectional associations between all individual determinants with dietary guideline adherence were separately assessed by linear regression models adjusting for age and sex. Effect modification for SEP was tested for each determinant via an interaction term. All results were stratified by SEP when at least one dietary behaviour variable showed a significant interaction with SEP or, in the case of non-significant interaction(s), the regression models were adjusted for SEP. Statistical significance was defined as *P* < 0·05. Quantitative analyses were conducted via R statistical software via the built-in *lm()* function and the *olsrr* package for residual diagnostics^(28)^.

#### Qualitative data analysis

All interviews were transcribed verbatim, and transcripts were imported in Atlas.ti for the qualitative data analysis^(29)^. The analysis was approached via a directed content analysis, according to an iterative process and semi-open coding technique^(30)^. Transcripts were read closely to become familiar with the content. An initial codebook was constructed as a starting point to code all interviews. This initial codebook was based on a conceptual framework describing the three elements of dietary behaviours (i.e. food choices, eating behaviour and dietary intake) as main themes. These three main themes included a number of pre-defined relevant sub-themes, based on the definition of dietary behaviours by Stok *et al.* ^(7)^ and for which quantitative and/or qualitative data were collected (online Supplementary Table 4). Emerging themes and sub-themes were further specified through open coding and a constant comparative analysis.

Analyses were initially conducted by JMS, a registered dietitian and predoctoral research fellow in the field of nutritional epidemiology, with training and experience in qualitative research methods. The first six interviews were (sub-)coded based on the initial codebook and open-end coding. After coding the first six interviews, all codes were critically examined and revised where necessary. The next six interviews were coded using this codebook, while changes in the codebook were logged during the coding process. Again, codes were critically examined and revised where necessary. This process was repeated until all interviews were coded. As a means of triangulation, JDM independently coded three interviews using the initial codebook. Coded themes and sub-themes in those interviews from JDM and from JMS were merged and compared in detail. Interpretation and formulation of all themes and sub-themes were discussed, and the codebook was revised accordingly. Next, JDM independently coded another four interviews, and these themes and sub-themes were again merged and compared and discussed in detail until consensus on coding was reached and a pre-final codebook was established. JMS re-evaluated all coding of the remaining seventeen interviews using the pre-final codebook. Ultimately, fifteen themes with 119 sub-themes emerged (online Supplementary Table 5), among which 100 % of final themes and 97 % of the final sub-themes were identified in the pre-final codebook indicating data saturation was accomplished.

#### Integrated data analysis

To identify how quantitative and qualitative data on adherence to dietary guidelines correspond and complement each other, we compared quantitative scores on the fifteen food group components of the DHD15-index for each individual interviewee to their own statements about these components in the qualitative interviews (*n* 24). We report to what extent the two data sources correspond (i.e. to what extent quantitative intake scores match findings from qualitative data), and whether there were notable details interviewees provided in the interviews about the nature of their dietary intake (e.g. vegetable consumption consisted of only one type of vegetable). The second objective focussed on identifying similarity in the determinants of dietary guideline adherence between data sources and across interviewees with varying socio-economic backgrounds. Therefore, we present quantitative associations between the determinants of dietary guideline adherence and total dietary guideline adherence scores (*n* 1492). Subsequently, we explored if these determinants were also important themes in the qualitative interviews, if there were notable differences in findings between interviewees with varying socio-economic backgrounds and if there were important themes derived from the interviews which were not captured by the quantitative data (*n* 24). The third objective aimed to identify interdependence among the determinants of dietary guideline adherence. As such, based on co-occurrence of codes and themes in the qualitative interviews (*n* 24), we explored the interdependence among the determinants of dietary guideline adherence.

## Results

### Study population characteristics

Participants within the quantitative sample (*n* 1492) were on average 42·4 (±13·7) years of age, 67·0 % was female and 45·4 % was categorised as having a high SEP. The total dietary guideline adherence score was on average 95·9 (±18·5) points on the DHD15-index (Table 1). Among the qualitative sample, which was used for the integrated analyses (*n* 24), interviewees were on average 50·6 (±10·3) years of age, 62·5 % was female and 16·7 % was categorised as having a low SEP, 58·3 % as medium SEP and 25·0 % as high SEP.

**Table 1:** Study population characteristics and the questionnaire scores of the quantitative data from the *Eet & Leef* study (*n* 1492)

Population characteristics	Total sample (*n* 1492)	
Female, *n* (%)	999	67·0
Age in years, mean (sd)	42·4	13·7
Socio-economic position, *n* (%)		
Low	257	17·2
Medium	557	37·3
High	678	45·4
Dietary guideline adherence (adherence score range from low to high)		
Total DHD15-index (1–150), mean (sd)	95·9	18·5
Vegetables (1–10), mean (sd)	6·3	3·1
Fruits (1–10), median (IQR)	6·9	5·9
Whole grains (1–10), mean (sd)	6·7	3·0
Legumes (1–10), median (IQR)	10·0	7·8
Nuts (1–10), median (IQR)	2·9	9·0
Dairy products (1–10), median (IQR)	4·7	5·8
Fish (1–10), median (IQR)	5·0	5·0
Tea (1–10), median (IQR)	3·3	9·8
Filtered coffee (1–10), mean (sd)	6·9	2·9
Oil and fat (1–10), median (IQR)	10·0	9·7
Red meat (1–10), mean (sd)	9·3	2·1
Processed meat (1–10), median (IQR)	6·4	6·8
Sugar-sweetened beverages (1–10), mean (sd)	7·3	3·5
Alcohol (1–10), mean (sd)	7·7	3·6
Salt (1–10), mean (sd)	8·2	2·2
Determinants of dietary guideline adherence (construct score range from low to high)		
Frequency of home cooking (1–13), mean (sd)	11·5	2·0
Frequency of not home cooking (1–13), mean (sd)	3·1	1·3
Grocery shopping style (1–5), mean (sd)	3·4	0·9
Consumption of snacks (1–7), mean (sd)	3·7	1·1
Cooking skills (1–5), mean (sd)	4·0	0·8
Sour taste preference (1–5), mean (sd)	3·0	0·9
Salt taste preference (1–5), mean (sd)	3·6	0·9
Sweet taste preference (1–5), mean (sd)	3·6	0·9
Habit strength vegetable consumption (1–7), mean (sd)	5·8	1·3
Cognitive restraint of eating (1–4), mean (sd)	2·3	0·6
Uncontrolled eating (1–4), mean (sd)	2·1	0·6
Emotional eating (1–4), mean (sd)	2·1	0·9
Parental upbringing regarding dietary habits* (1–2), mean (sd)	1·7	0·2

sd, standard deviation; IQR, interquartile range; Supplementary Table 2 presents interpretation of score ranges.

* *n* = 337 missing values.

### Objective 1: correspondence and complementarity of data sources on dietary intake

When interviewing about a habitual intake patterns, interviewees frequently mentioned the consumption of vegetables, fruits, dairy and red meat during the interviews. Some interviewees mentioned fish, tea, coffee, processed meats, sugar-sweetened beverages and alcoholic beverages, and only a few mentioned whole grains, legumes, nuts, oils and fats, and salt. There was a strong correspondence between the interviewees’ own quantitative and qualitative data for the components dairy, fruits and red meat. However, contradictory findings emerged for vegetables, predominantly among interviewees with a low SEP and a high SEP. A number of interviewees who did not mention vegetables generally had lower adherence scores for vegetable intake, but many other interviewees indicated to perceive vegetables as highly important and/or consume vegetables as part of their dietary routine while they scored relatively low on vegetables intake.

‘*I need to have my daily portion of fruits. And my vegetables. That’s just how I always do it*’. (Low SEP female #3 who scored ‘4’ on vegetables)

Concerning red meat, high adherence scores (i.e. low red meat consumption) were aligned with qualitative findings in interviewees from both a low SEP and a high SEP. For example, vegetarian patterns, deliberately trying to reduce meat consumption, aversion against mouthfeel of meat structure or early experienced satiety after meat consumption. Interviewees who had high adherence scores for fruit intake mentioned to consume fruits as part of their dietary routines, while other interviewees who had lower adherence scores indeed stated to not consume fruits on a regular basis as illustrated by the quote below. Interviewees with a medium SEP rarely mentioned fruit and red meat consumption.

*‘I try to eat as healthy as possible, including vegetables and such. I don’t always manage to eat fruits, but coincidentally did last week’.* (Low SEP female #1 who scored ‘1’ on fruits)

Overall, dairy consumption by interviewees with varying socio-economic backgrounds corresponded to their quantitative adherence score. Most interviewees who had relatively low adherence scores for dairy mentioned to eat cheese but not any other dairy products; they mentioned to consume a single portion of daily dairy (e.g. at breakfast or as dessert) or to not consume milk at all. Those with high adherence scores for dairy indeed indicated to consume multiple dairy portions.

### Objective 2: Similarity in the determinants of dietary guideline adherence between data sources

None of the determinants of dietary guideline adherence showed significant interaction (*P* < 0·05) with SEP. Therefore, Table 2 presents the associations between determinants of dietary guideline adherence and total adherence scores, adjusted for sex, age and SEP. Qualitative data provided insights in how determinants affected dietary guideline adherence across individuals with varying socio-economic backgrounds. For some of the determinants, variation emerged between interviewees with different SEP which are reflected upon below.

**Table 2:** Cross-sectional associations between determinants of dietary guideline adherence and total Dutch dietary guideline adherence scores (DHD15-index) among a Dutch population sample (*n* 1492)

	Total sample (*n* 1492)	
	*β*	95 % CI
Frequency of home cooking	**1·5**	**1·0, 1·9**
Frequency of not home cooking	**−2·3**	**–3·0, –1·6**
Grocery shopping style	0·7	–0·3, 1·7
Consumption of snacks	−0·8	–1·5, 0·0
Cooking skills	**4·7**	**3·5, 5·9**
Sour taste preference	**1·3**	**0·3, 2·4**
Salt taste preference	0·1	–0·9, 1·1
Sweet taste preference	0·6	–0·4, 1·5
Habit strength vegetable consumption	**4·0**	**3·3, 4·7**
Cognitive restraint of eating	**5·6**	**4·2, 7·1**
Uncontrolled eating	0·8	–0·8, 2·4
Emotional eating	0·4	–0·7, 1·4
Parental upbringing regarding dietary habits*	**6·0**	**1·9, 10·2**

Supplementary Table 2 presents interpretation of score ranges; bold values represent statistically significant findings (*P* < 0·05). All associations were adjusted for age, sex and socio-economic position.

* *n* = 337 missing values as a result of answer category ‘I do not know’.

#### Frequency of (not) home cooking

Higher frequency of home cooking was associated with higher dietary guideline adherence (*β* 1·5, 95 % CI 1·0, 1·9), and higher frequency of not home cooking was associated with lower dietary guideline adherence (*β* –2·3, 95 % CI –3·0, –1·6) (Table 2). Qualitative data revealed that most interviewees with a medium and high SEP noted to order take out or go out for dinner approximately once every 2 weeks to once a month, mostly as a social activity with the family or when they did not feel like cooking. On the other hand, interviewees with a lower SEP often mentioned that going out for dinner was a special event for which money should be saved up front, or which was for example considered challenging in terms of choosing a restaurant due to perceived fussy eaters in the family.

*‘When I go out for dinner with my family, we can only go stir fry restaurants. Because they are such whiners; they only want to eat stir fry’.* (Low SEP female #3 who scored ‘115’ on total dietary guideline adherence)

#### Cooking skills

Better cooking skills were associated with a higher dietary guideline adherence (*β* 4·7, 95 % CI 3·5, 5·9) (Table 2). Qualitative data indicated that cooking skills were an important determinant across nearly all interviewees. Cooking was generally viewed as a necessity which was by most experienced as an enjoyable activity to put time and effort in, but for some as a necessary obligation. For example, interviewees explained to have learned cooking from cookbooks or dinner boxes including recipes and ingredients, and some indicated to have allocated financial resources to purchase special kitchen equipment.

*‘I am a fan of cooking myself. I also bought some cookbooks and special pots and pans’.* (Low SEP male #1 who scored ‘82’ on total dietary guideline adherence)

#### Habit strength related to vegetable consumption

Higher habit strength related to vegetable consumption was another determinant for higher dietary guideline adherence (*β* 4·0, 95 % CI 3·3, 4·7) (Table 2). From the qualitative data, habit strength related to vegetables emerged predominantly among interviewees with a medium and a high SEP. They mentioned a range of habits, such as eating vegetables during lunch on a daily basis, adhering to vegetable recommendations during holidays, consuming personally disliked vegetables (e.g. Brussels sprouts), first consuming vegetables during diner, frequently visiting greengrocers or street markets to purchase many vegetables or keeping in mind if the daily recommended amount of vegetables is reached.

#### Cognitive restraint of eating

Higher levels of cognitive restraint of eating were also a determinant of dietary guideline adherence, as higher levels of cognitive restraint were associated with a 5·6 point (95 % CI 4·2, 7·1) higher adherence (Table 2). The qualitative data provided insight into ‘self-imposed rules’ of interviewees, such as ensuring to not have unhealthy foods at home, avoiding exposure to purchase occasions of unhealthy foods (e.g. snack bar), eating smaller portions, allowing a certain daily maximum of a certain unhealthy products or eliminating certain unhealthy products completely (e.g. crisps). In addition, especially interviewees with a lower SEP specifically mentioned to deliberately restrict their dietary intake to avoid weight gain.

*‘If I am getting a bit too heavy, I notice that I have to reduce it [food intake] a bit and then I manage’.* (Low SEP female #1 who scored ‘79’ on total dietary guideline adherence)

#### Parental upbringing

Stricter parental upbringing regarding dietary habits showed the largest association with higher dietary guideline adherence (*β* 6·0, 95 % CI 1·9, 10·2) (Table 2). Qualitative data revealed that interviewees mostly described their parental upbringing as ‘normal’. Their perception was to have learned to eat healthy products and to eat whatever is provided. For example, most interviewees noted that vegetables were obligatory during diner and some indicated that they only received candy during the weekend.

#### Sour taste preference

Sour taste preference was associated with higher dietary guideline adherence (*β* 1·3, 95 % CI 0·3, 2·4) (Table 2), but sour taste preferences as a relevant determinant for dietary guideline adherence did not emerge from the interviews. Yet, a variety of other determinants of dietary guideline adherence were identified from the qualitative data and notable findings are described below.

#### Other determinants identified from the qualitative data

Food prices emerged as relevant determinant for dietary guideline adherence among nearly all interviewees with a low and a medium SEP, while less among those with a high SEP. Especially, the high price of fruits and vegetables was frequently mentioned affecting purchase decisions and thus the frequency of consuming these products and the variety of types of products consumed. Also, interviewees perceived that cooking at home is more value for money, and that checking of price promotions was a common habit and for some a reason to visit a certain supermarket.

*‘Last weekend I wanted to prepare a cauliflower dish, but I noticed that a cauliflower was 2 Euros. Well, never mind then’.* (Low SEP female #1 who scored ‘86’ on total dietary guideline adherence)

Strong dietary habits emerged from almost all interviews as a determinant affecting dietary guideline adherence. Most interviewees mentioned following highly similar meal patterns daily, where a number of interviewees noted that structure and type of meals were focussed on providing consistency for their children. For some, their strong dietary habits were perceived as a barrier to improve their dietary intake (e.g. snack-related habits), while for others their strong habits helped to adhere to a healthy meal pattern.

*‘I just prepare my breakfast and lunch in the morning, even on weekends when I don’t have to leave to house. Then I only have to prepare my bread just once and I can just grab it when I’m hungry: low-carb bread with peanut butter and cucumber’. (High SEP male #1 who scored ‘121’* on total dietary guideline adherence)

The social aspect of eating emerged as a positive determinant as well. Interviewees indicated that eating at home with their family during dinner time was a moment to socially connect, and some enjoyed cooking or baking for family, friends, neighbours and colleagues. Transitions in dietary habits motivated by health-related factors emerged as a determinant of dietary guideline adherence mostly among interviewees with a higher SEP: prevalent overweight and the desire to age healthily were frequently mentioned as reason to shift towards healthier dietary habits.

Importance of pleasure of eating emerged as a relevant determinant for some interviewees and mostly among those with a low and medium SEP. Interviewees emphasised that the taste of food is highly important and in some cases more important than the healthiness of products.

*‘When it comes to food, I just don’t want to put any restrictions on myself. Food is just way too tasty’.* (Medium SEP female #9 who scored ‘67’ on total dietary guideline adherence)

### Objective 3: Interdependence among the determinants of dietary guideline adherence

The qualitative interviews revealed that determinants clustered together in various ways (Table 3).

**Table 3:** Overview of the four clusters of interdependent determinants of dietary guideline adherence as emerged from the qualitative data

Cluster 1	Cluster 2	Cluster 3	Cluster 4
Health-related considerations	Health-related considerations	Snack consumption	Negative attitude towards cooking and food-related activities
Perceived healthiness of dietary intake	Cooking skills	Cognitive restraint	Preferences of other family members
Cooking skills	Snacking behaviour	Self-imposed rules	
Self-imposed rules	Uncontrolled emotional eating	Aiming to lose weight	
Strong dietary habits	Social aspect of eating	Avoiding sugar and/or carbohydrates	
Social aspect of eating			

First, the data suggested that the sub-themes health-related considerations, perceived healthiness of the dietary intake, cooking skills, self-imposed rules, strong dietary habits and the social aspect of eating were interdependent in their relation with dietary guideline adherence. For example, an interviewee explained that she adhered to similar dietary patterns during holidays as during regular working weeks (strong dietary habits), albeit with some variation in the level of difficulty of dishes (cooking skills), to be conscious about the healthiness of dietary choices, for example including plenty of vegetables (perceived healthiness of dietary intake), to limit meat intake and to abstain from sauces considering those were perceived as unhealthy causing weight gain (self-imposed rules and health-related considerations). Furthermore, a number of interviewees indicated that cooking a fresh meal without, for instance, the use of pre-packaged seasoning mixes was perceived as healthy and more satiating. It motivated them to put in effort via the use of recipes, searching for nutrition information on the Internet and reading of nutrition labels. A number of interviewees indicated that wanting to provide healthy food for children was a key motivator to invest time and effort in cooking nutritious meals and to deliberately limit the use of take-out meals (e.g. by preparing homemade pizza). Health-related considerations also occurred in combination with strong dietary habits and cooking skills, for example, by cooking at home as much as possible and by limiting pre-packaged products or ingredients (e.g. sugar and salt) which were perceived as unhealthy.

*‘I believe it is highly important to use fresh products when preparing dinner. So, no pre-packaged products with additives. Actually, I never use cream or anything like that. And when I use it, I use a third of what the recipe indicates. I’m not saying I never eat it, but I do try to be very conscious. I believe you can just prepare home-made pasta sauce. Because of this rule for myself, I think we eat healthy’.* (High SEP female #1 who scored ‘95’ on total dietary guideline adherence)

Second, health-related considerations and cooking skills also emerged in combination with snacking behaviour, uncontrolled emotional eating and the social aspect of eating. Some interviewees indicated knowing what a healthy diet is composed of and how to cook healthy meals. At the same time, they found it highly challenging to resist unhealthy snacks. Two major determinants of snacking behaviour emerged. First, for some interviewees, snacking behaviour was the result of emotional eating when feeling down (emotional eating).

*‘So, if it’s not going so alright with me, then I’m going to eat. Then I will eat unhealthy things. I am going to eat really bad things, and I cannot stop it’.* (Medium SEP female #3 who scored ‘98’ on total dietary guideline adherence)

This snacking behaviour was frequently accompanied by experienced shame and the desire to hide such behaviour from their social environment. Being accompanied by family (social control) actually helped to limit snack intake. Contradictorily, the social aspect of eating also emerged as the second determinant of snacking behaviour: exposure to snack foods together with friends and family actually facilitated overconsumption for some interviewees, where others experienced social support from their family in not consuming snack foods within their household setting. Moreover, food preferences of others in the household related to snack foods were a social barrier for a healthy dietary intake.

Third, snack consumption also emerged in combination with cognitive restraint, self-imposed rules, aiming to lose weight and avoiding sugar and/or carbohydrate intake. One interviewee explained to have a rule for snacking during parties: only consume the cheese, without the toast. Others avoided all snacks (e.g. crisps) or all carbohydrates including fruits, considering limiting sugar and/or carbohydrates was perceived as healthier and easier to adhere to than low-calorie diets and to help with weight loss.

Fourth, a few interviewees noted a negative attitude towards cooking and food-related activities due to preferences of other family members with specific dietary wishes, sometimes perceived as fussy eaters. For example, some interviewees cooked different types of meals on a single night (e.g. whole-grain pasta and regular pasta) while others never bought certain types of vegetables or other products as a result of preferences of family members.

*‘I do not always cook with pleasure, because sometimes you just do not know anymore what to prepare for the family. Look, if you lived alone, it’s very different. Then you can just prepare something quickly. And also a bit healthier. Now I have to think about my daughter, but I also have to think about my husband. What they want to eat and what they like, and that can change by the day. I find it mentally exhausting because you just do not know what to prepare’.* (Medium SEP female #8 who scored ‘64’ on total dietary guideline adherence)

## Discussion

With this mixed-methods study, we explored to what extent quantitative measurements of dietary guideline adherence and its determinants reflect the perceptions of adults with varying socio-economic backgrounds thereof. Quantitative and qualitative data on dietary guideline adherence emerged as corresponding for most food groups but seemed contradictory regarding vegetable consumption. Results suggested a misconception on adequate vegetable intake, predominantly among interviewees with a low and a high SEP. Furthermore, important determinants of higher dietary guideline adherence as derived from quantitative data were higher frequency of home cooking, better cooking skills, higher levels of habit strength related to vegetables, higher levels of cognitive restraint of eating and stricter parental upbringing regarding dietary habits – all without socio-economic differences in the importance of these factors for guideline adherence. These determinants also emerged from the qualitative data, which provided insight into the way in which the determinants affected dietary guideline adherence. Qualitative data suggested additional determinants for guideline adherence, with food prices, strong dietary habits and the social aspect of eating most prominently featured. Some variation between interviewees with different SEP emerged from the qualitative data in how the determinants of cognitive restraint, habit strength related to vegetables, food prices and home cooking affected dietary guideline adherence. There was a notable interdependence of determinants for guideline adherence, most evident for the interdependence of health-related considerations, perceived healthiness of dietary guideline adherence, cooking skills, self-imposed rules, strong dietary habits and the social aspect of eating.

Interviewees’ quantitative scores on dietary guideline adherence largely reflected their perceived food intake, except for vegetable intake. The observation that predominantly interviewees from both low and high SEP perceived their vegetable intake to be higher than the quantitative intake scores on vegetables is not fully consistent with previous literature. Previous studies suggest that overestimation of own vegetable intake is more present among individuals with lower educational levels^(31,32)^. Unawareness of dietary recommendations or, if aware, being uninterested in recommendations could explain the overestimation of own intake. Also, the presence of optimistic bias in the self-assessment of dietary intake levels is well known, and the perception of own intake, the comparison to the average intake of others and the comparison to dietary guidelines may all affect the perceived vegetable intake^(33,34)^. In all cases, a mismatch between the perceived *v*. actual vegetable intake can inhibit diet modifications among individuals and attenuate effects of nutritional interventions promoting vegetable consumption^(34)^. Therefore, new interventions focusing on the promotion of vegetable consumption should consider awareness on sufficient vegetable consumption and study if overestimation of own intake can be reduced.

Our quantitative data revealed no socio-economic differences in determinants of dietary guideline adherence, while our qualitative data provided a richer understanding in how these determinants contributed to dietary guideline adherence across interviewees with varying socio-economic backgrounds. Food preparation behaviours (cooking at home *v*. and not cooking at home) were associated with dietary guideline adherence for all socio-economic groups, while food shopping behaviours were not. Yet, qualitative data suggested variations between interviewees with different SEP in the frequency of (not) home cooking, as those with lower SEP and some with medium SEP indicated having limited financial resources to spend on eating out. A previous observational study from France reports that those with lower SEP generally rely on less kitchen equipment but are investing more time in cooking when compared to higher SEP^(12)^. Notably, quantitative data thereby indicated that higher frequency of home cooking and better cooking skills were associated with higher dietary guidelines adherence. A previous study based on the *Eet & Leef* study found that 13–19 % of the relationship between SEP and the DHD15-index was explained by cooking skills^(11)^. Findings from the present study suggest that interviewees with a lower SEP were used to home cooking, and perhaps they have other cooking skills than quantified via the questionnaire items. For example, a number of them described limiting the use of pre-packaged products and they may have good cooking skills with limited cooking equipment. Moreover, perceptions relating to cooking and cooking skills were associated with positive and also negative attitudes towards food preparation. Some interviewees enjoyed cooking while others saw it as a necessary or even difficult task. Notably, in a number of cases when talking about home cooking, interviewees spoke negatively about their meal planning due to the influence of family members with very specific taste preferences. As a solution, some cooked different food options per meal. Others struggled with meal planning aiming to keep every family member satisfied, something that is reflected by previous literature^(35,36)^.

Habit strength of vegetable consumption was mainly mentioned by interviewees with a medium and a high SEP. They referred to a range of habits (e.g. eating vegetables during lunch, adhering to vegetable recommendations during holidays, or consuming personally disliked vegetables) related to vegetable intake, suggesting a more comprehensive skill set for enhancing vegetable consumption than those with lower SEP. Low SEP interviewees mostly referred to purchasing habits related to vegetables but no other habits, and limited financial resources often emerged as a determinant inhibiting vegetable (and fruit) purchasing. The high price of vegetables is a well-known barrier for sufficient vegetable consumption, as well as overall higher prices of healthy foods compared to unhealthy foods^(37)^.

Regarding cognitive restraint of eating, it was notable that specifically those with a lower SEP mentioned to restrict their intake to avoid weight gain when they felt this was necessary, while interviewees with medium and higher SEP spoke of a greater variety of strategies incorporated in their daily routines to secure healthier dietary choices. As such, it might be that those with medium and higher SEP generally implemented more sustainable habits, whereas those with lower SEP might rely on incidental periods of restricted intake for weight management. This hypothesis builds upon previous literature in which stronger levels of cognitive restraint were observed for those with a higher SEP (e.g.^(38)^).

Lastly, quantitative data also indicated that perceptions of stricter parental upbringing regarding dietary habits were associated with better adherence to dietary guidelines. Strictness in parental rules around eating was not mentioned specifically by the interviewees. Among almost all interviewees, there was a strong perception of a ‘normal’ upbringing which may refer to perceived stability and consistency in the dietary upbringing, which could facilitate formation of healthier habits and ultimately contribute to higher guideline adherence as an adult.

Some additional determinants of dietary guideline adherence emerged from the qualitative data which were not captured in the quantitative data. For many interviewees, taking into account the family preferences and structure and consistency in meal planning for children resulted in strong dietary habits. For some, these were healthy habits, but for others family preferences were perceived as problematic in meal planning. Across socio-economic groups, a number of parents were uncertain about what to prepare and some cooked various types of meals which met preferences of all family members. Others relied on their own strong healthy habits which already existed before having children, and they reported experiencing no difficulties with meal planning within their family. As such, strong healthy habits with resilience to the social environment appeared to enable higher dietary guideline adherence. Furthermore, for some interviewees, their own strong dietary habits were a facilitator in healthy eating (i.e. no internal debates on whether or not to choose healthy options), while for others they were a barrier to change their dietary intake (i.e. automatically turning to unhealthy choices). In addition, social support from a partner in healthier choices is described as a facilitator for healthier diets^(35)^, a finding which also emerged from our qualitative data. Some interviewees reduced snacking behaviours in the presence of partners who abstained from snacks. However, when they were alone they experienced difficulties to resist snacks, indicating that the presence of a partner is also a form of social control helping to eat healthier. These findings combined emphasise the relevance for developing strong healthy dietary habits and of the consideration of the social environment in relation to food decisions, home cooking and ultimately dietary guideline adherence.

As already became apparent from the previous paragraphs, the qualitative data revealed an interdependence between various determinants of dietary guideline adherence. The interplay between health-related considerations, cooking skills, snacking behaviour, emotional eating and the social aspect of eating was notable. Some interviewees indicated that knowledge on healthy food and food preparation did not automatically lead to a healthy dietary intake. Indeed, nutrition knowledge is only one of many determinants of dietary guideline adherence. The present findings indicate that the social environment is likely as important in either facilitating unhealthy food consumption (e.g. having many snacks at birthdays is the accepted social norm) or preventing overconsumption (e.g. when a partner is disapproving snack foods). Observations from our study add to a recent scoping review mapping the meaning of eating and motivations for healthy eating specifically among those with a low SEP, where social influences on food and eating practices emerged as one of the most relevant motivators for dietary choices – along with time and financial constraints, importance of parental upbringing and food traditions, and regulation of children’s diets^(36)^.

### Implications

Findings of this study improve our understanding of determinants of dietary guideline adherence among adults with varying socio-economic backgrounds within a real-life context. The insights from this study can be used to tailor nutrition interventions across populations when aiming to improve dietary behaviours in the context of public health interventions and ultimately to reduce nutrition-related health inequalities. Future nutrition interventions should target the formation of new (healthier) dietary habits which can be easily incorporated into daily routines, taking into account family and wider social circumstances. Furthermore, the insights on individual-level determinants of dietary guideline adherence should be considered within their broader context also driving dietary guideline adherence (i.e. the social–cultural, physical, economic and policy environments^(39)^). The current study points towards an urgent need to address the economic environment (i.e. addressing food prices of healthy products), but multi-level approaches, including individual-level approaches and environmental-level approaches, are crucial when aiming to improve dietary guideline adherence across populations. For example, there is tentative evidence that food environment (e.g. higher exposure to unhealthy food outlets in the neighbourhood) plays a role in dietary guideline adherence^(40)^, with possible interaction by SEP^(41)^. Fruit and vegetables programmes at schools (i.e. the social–cultural environment) may help to improve cultural norms on vegetable consumption among children, which for some households may facilitate greater social support in dietary choices and meal planning within families. Future research should seek to expand the knowledge on the complexity of determinants of dietary behaviours across populations with varying socio-economic backgrounds and investigate it from a broader perspective, for example, a socio-ecological perspective or a systems approach^(6,42)^.

### Strengths and limitations

The mixed-methods approach is a strong methodological aspect of this study, as it systematically integrated and enriched both types of data sources. It combined quantitative associations in a general population with in-depth views on the various aspects of dietary behaviours and their interplay across adults with varying socio-economic backgrounds. Furthermore, the quantitative sample consisted of a large group derived from various urban areas across the Netherlands, securing high generalisability for the Dutch population living in urban areas. Yet, it should be acknowledged that excluding individuals who did not understand the Dutch language and did not have access to a computer and e-mail address might have introduced some selection bias, leading to an underrepresentation of those with low (digital) literacy. Another limitation is the fact that the *Eet & Leef* study was not set up as a mixed-methods design. As such, some of the additional emerged qualitative determinants on dietary guideline adherence may have emerged from the quantitative data as well if items on these topics would have been included (e.g. food prices and social aspect of eating). Moreover, the used Dutch Healthy Diet FFQ is considered an acceptable instrument to rank individuals in dietary guideline adherence, but it performs relatively poor at individual assessment of absolute intake^(24)^. This should be considered for the interpretation of results at the first objective, where we compared individual scores to population averages. In addition, the use of telephone interviews was necessary as interviewees lived across the Netherlands, but it might have limited the opportunity to build rapport with interviewees. It potentially has affected the willingness of participants to share certain views. Also, being interviewed by public health researchers may have caused some interviewees to provide socially desirable answers relating to (healthier) dietary choices. On the other hand, many interviewees talked about facing challenging in making healthy choices, suggesting they felt comfortable in sharing their views and that socially desirable answers were limited.

## Conclusion

This mixed-methods exploration provides a richer understanding of why adults with varying socio-economic backgrounds do or do not adhere to dietary guidelines. Both quantitative and qualitative data showed similar determinants for higher dietary guideline adherence such as higher levels of cognitive restraint of eating, higher habit strength related to vegetable consumption, better cooking skills and frequency of home cooking. Qualitative but not quantitative data suggested socio-economic variations in how determinants attributed to higher guideline adherence, as cognitive restraint and habit strength of vegetables seemed more important determinants for interviewees with a higher SEP, while food prices and home cooking seemed more important for interviewees with a lower SEP. Our findings demonstrate that data sources on dietary guideline adherence complement each other and results can guide future interventions focussing on the promotion of healthy dietary patterns across populations with varying socio-economic backgrounds.

## Supplementary material

For supplementary material accompanying this paper visit https://doi.org/10.1017/S1368980023000228. click here to view supplementary material
