# Supplementary material for: Better understanding determinants of dietary guideline adherence among Dutch adults with varying socio-economic backgrounds through a mixed-methods exploration
Source: Public Health Nutr. 2023 Jan 26;26(6):1172–84. doi: 10.1017/S1368980023000228 (PMC10348427; doi:10.1017/S1368980023000228)
Supplement: Supplementary file 1 [file S1368980023000228sup001.docx]

**Better understanding determinants of dietary guideline adherence among Dutch adults with varying socioeconomic backgrounds through a mixed-methods exploration**

Supplementary material

J. M. Stuber, *et al*

#

**Supplementary table 1**. Descriptive statistics on summary scores of the SEP indicators income, education and occupation (n=1,492)

|  | **Income^a^** | **Education^b^** | **Occupation^c^** |
| --- | --- | --- | --- |
| Low, *n* (%) | 368 (26,8) | 150 (10,1) | 46 (3,3) |
| Medium, *n* (%) | 795 (57,9) | 482 (32,6) | 436 (31,3) |
| High, *n* (%) | 210 (15,3) | 848 (57,3) | 911 (65,4) |

SEP = Socioeconomic position. ^a^*n*=119 missing values; ^b^*n*=12missing values; ^c^*n*=99 missing values.

# **Supplementary table 2.** Description of quantitative variables and their score interpretation

|  | Shortened description of questionnaire items | Questionnaire item source and validity | Interpretation of summary score |
| --- | --- | --- | --- |
| Dietary guideline adherence | 34-item food frequency questionnaire including questions on the intake of the following dietary components:   1. Vegetables; 2. Fruit; 3. Whole grain products; 4. Legumes; 5. Nuts; 6. Dairy; 7. Fish; 8. Tea; 9. Fats; 10. Coffee; 11. Red meat; 12. Processed meat; 13. Sugary drinks; 14. Alcohol; 15. Salt. | Dutch healthy diet FFQ by van Lee et al. (2016)^(1)^:  This short screener questionnaire was validated against a 180-item FFQ combined with a 24h urinary Na excretion value.  Correlation between  the DHD-index derived from the DHD-FFQ and the reference method was 0.56 (95 % CI 0.52, 0.60).  The DHD-index derived from the DHD-FFQ was acceptable in ranking but relatively poor in individual assessment of diet quality. | Individual dietary components are scored 0-10 and the sum of these dietary components reflect the total diet quality scored 0-150.  Higher scores are associated with a higher adherence to the Dutch Dietary guidelines, i.e., a healthier dietary intake. |
| Frequencies of (1) home cooking and of (2) not home cooking | Frequency of:   1. Home cooking.   And frequency of not home cooking:   1. Ordering meals; 2. Eating out of home; 3. Preparing ready-to-eat meals. | N/A | 13-point scale (ranging from 1=never to 13=everyday):  Higher summary scores are associated with a higher frequency of (1) home cooking,  or of (2) not at home cooking. |
| Grocery shopping style | Frequency of:   1. Making a shopping list before doing groceries; 2. Purchasing groceries for the whole week at once; 3. Deciding during grocery shopping what to buy. | N/A | Five-point Likert scale: Higher summary score is associated with a less impulsive and more planned/organized shopping style. |
| Consumption of snacks | Frequency consumption:   1. Sweets; 2. Chocolate; 3. Cookies; 4. Cake or pie; 5. Ice cream; 6. Crisps or salty snacks; 7. Nuts; 8. Pizza; 9. Fried snacks; 10. Sausage rolls. | N/A | Seven-point Likert scale: Higher summary score is associated with more frequent snacking. |
| Cooking skills | 1. Able to prepare fresh vegetables in different ways; 2. Difficult to prepare a meal with more than five fresh ingredients; 3. Able to alter a recipe; 4. Able to prepare fresh fish in different ways; 5. Able to prepare a meal using fresh ingredients; 6. Able to see, smell or feel the quality of fresh foods. | Self-perceived food literacy scale by Poelman et al. (2018)^(2)^:  Confirmatory Factor Analysis could not confirm the proposed factor structure of the 6 items loading onto one factor (CFI = 0.97, RMSEA = 0.07, TLI = 0.95). Exploratory factor analysis suggested that one item needed to be removed and that the remaining five items loaded onto one factor. Internal consistency of those items was good (Cronbach’s alpha in this study: 0.77) and test-retest reliability was good (Spearman’s correlation of 0.84). | Five-point Likert scale: Higher summary score is associated with better cooking skills. |
| Taste preferences:  (1) Sour, (2) salt, and (3) sweet | Taste preference:   1. Sour; 2. Salt; 3. Sweet. | N/A | Five-point Likert scale: Higher summary scores are associated with a stronger taste preference for (1) sour, (2) salt, (3) sweet. |
| Habit strength vegetable consumption | Consuming vegetables:   1. Often; 2. Automatically; 3. Not conscious; 4. Odd not doing; 5. Without thinking; 6. Effort not doing; 7. Routine; 8. Begin automatically; 9. Difficult not doing; 10. Not thinking; 11. Typically me; 12. Doing for long time. | Self-report index of habit strength by Verplanken & Orbell (2003)^(3)^:  Confirmatory Factor Analysis could not confirm the proposed factor structure of the 12 items loading onto one factor (CFI = 0.87, RMSEA = 0.17, TLI = 0.84). However, exploratory factor analysis suggested that all 12 items load onto one factor. Internal consistency was excellent (Cronbach’s alpha in this study: 0.96) and test-retest reliability was questionable (Spearman’s correlation of 0.69). | Seven-point Likert scale: Higher summary score is associated with a stronger habit to consume vegetables. |
| Eating habits: (1) Uncontrolled eating, (2)  Emotional eating, and  (3) Cognitive restraint of eating. | 1. Eating small portions for weight control; 2. Deliberately limit food intake to prevent weight gain; 3. Avoiding some foods because they are perceived to make fat; 4. Hard not to eat tasty looking or smelling foods, even just after finishing a meal; 5. Always so hungry that stomach feels like a bottomless pit; 6. Always so hungry that it is hard to stop eating before the plate is empty; 7. Always hungry enough to eat at any time of the day; 8. When seeing something really tasty immediately getting so hungry that it has to be eaten 9. When with someone who eats often, also getting hungry enough to eat something as well; 10. Sometimes can't stop when starting eating; 11. Often eat too much when feeling unhappy; 12. Eating when worried; 13. Comforted by eating when feeling lonely; 14. Frequency of avoiding to hoard tempting foods; 15. Frequency of feeling hungry; 16. Frequency of eating too much even when not hungry; 17. Frequency of consciously eating less than desired; 18. Frequency of restraining from eating. | Three-Factor Eating Questionnaire Karlsson et al. (2000)^(4)^:  Exploratory Factor Analysis indicated that items 4, 10, 14 and 16 should be removed and that the remaining items load onto three factors.  Factor 1 (uncontrolled eating) items 5, 6, 7, 8, 9 and 15. Factor 2 (emotional eating) items 11, 12 and 13. Factor 3 (cognitive restraint) items 1, 2, 3, 17 and 18.  Internal consistency was good (Cronbach’s alpha factor 1: 0.86, factor 2: 0.93, and factor 3: 0.82). | Four-point Likert scale: Higher summary scores are associated with higher levels of uncontrolled eating, emotional eating, or cognitive restraint of eating (i.e., having control over food intake in order to influence body weight). |
| Parental upbringing regarding dietary habits | Agreed (yes), or not agreed (no), or (I do not know):   1. Used to eat diner in front of the television; 2. Parents used to have strict rules about bread toppings; 3. (Almost) always had breakfast together with the family; 4. Used to always have to empty the diner plate; 5. Used to usually eat dessert after dinner; 6. Parents had rules about a maximum number of sweets / cookies per day; 7. Used to only get soft drinks at a party or special occasion. | N/A | Higher summary score is associated with a more strict parental upbringing regarding dietary habits.  The answer category ‘I do not know’ was treated as a missing value as there is no reliable score to assign to this answer category. |

N/A: Not applicable.

(1) van Lee L, et al. (2016) Evaluation of a screener to assess diet quality in the Netherlands. Br J Nutr 115, 517-526.

(2) Poelman MP, et al. (2018) Towards the measurement of food literacy with respect to healthy eating: the development and validation of the self-perceived food literacy scale among an adult sample in the Netherlands. Int J Behav Nutr Phys Act 15, 54.

(3) Verplanken B, Orbell S (2003) Reflections on past behavior: A self-report index of habit strength. J Appl Soc Psychol 33, 1313-1330.

(4) Karlsson J, et al. (2000) Psychometric properties and factor structure of the Three-Factor Eating Questionnaire (TFEQ) in obese men and women. Results from the Swedish Obese Subjects (SOS) study. Int J Obesity 24, 1715-1725.

# **Supplementary table 3**. Summary semi-structured interview guide qualitative study.

| Introduction: |
| --- |
| 1 Aims and structure of the interview |
| 2 Participants’ own description of their dietary habits (e.g., typical week and exceptions) |
| First part: Dietary behaviors |
| 3 Transitions in dietary habits (e.g., in adolescence, after marriage) |
| 4 Variations in dietary habits (e.g., weekdays vs. weekend days, with vs. without company) |
| 5 Individual characteristics related to dietary habits (e.g., shopping style, giving in to temptations) |
| 6 Role of food in life (e.g., does it give pleasure, link with health) |
| Second part: Role of the food environment |
| 7 Locations to buy food and reasons for choosing these locations |
| 8 Opinion on availability, accessibility and affordability of health and unhealthy foods |
| 9 Whether the food environment and broader neighborhood has changed in the last years |
| Final part: |
| 10 Own barriers and facilitators to healthy eating in the food environment |
| 11 Others’ barriers and facilitators to healthy eating in the food environment |
| 12 Any other questions/remarks and closing |

# **Supplementary Table 4.** Initial codebook with pre-defined themes and sub-themes for the qualitative analysis

| **Dietary behavior category** | **Theme** | **Sub-theme** |
| --- | --- | --- |
| Food Choices | Food preferences | Taste preference (salt/sweet/sour) |
|  |  | Health-related concerns |
|  |  | Food quality |
|  | Grocery shopping style |  |
|  | Income (to) spent on food/willingness-to-pay |  |
|  | Food preparation | Cooking skills |
|  |  | (Frequency of) Home cooking |
|  |  | Ordering food/ going out for diner |
| Eating Behaviors | Eating habits | Uncontrolled and emotional eating |
|  |  | Cognitive restraint of eating |
|  | Eating occasions (e.g. frequency/time) |  |
|  | Portions (number/size) |  |
|  | Parental upbringing regarding dietary habits |  |
|  | Transitions in dietary habits (e.g., in adolescence, after marriage) |  |
|  | Habit strength related to vegetable consumption |  |
|  | Consumption of snacks |  |
|  | Dieting |  |
| Dietary Intake | Dietary pattern | Type of pattern |
|  |  | Variation in pattern |
|  | Overall dietary quality |  |
|  | Dietary components | Vegetables |
|  |  | Fruits |
|  |  | (Whole) grains |
|  |  | Legumes |
|  |  | Nuts |
|  |  | Dairy |
|  |  | Fish |
|  |  | Tea |
|  |  | Coffee |
|  |  | Fats |
|  |  | Red meat |
|  |  | Processed meat |
|  |  | Sugar sweetened beverages |
|  |  | Alcohol |
|  |  | Salt |

# **Supplementary Table 5.** Final codebook for the qualitative analysis

| **Dietary behavior category** | **Theme** | **Sub-theme** |
| --- | --- | --- |
| Food Choices | Food preferences | Sweet taste preference |
|  |  | Salt/savory taste preference |
|  |  | Preferring all kinds of flavors/foods |
|  |  | Health-related considerations |
|  |  | No health-related considerations |
|  |  | Healthy food for children |
|  |  | Food quality/ freshness/organic |
|  |  | Minimally processed foods |
|  |  | Food aversions |
|  |  | Convenience |
|  |  | New foods/ recipes at home |
|  |  | Enjoying new foods/recipes when eating out |
|  |  | Not enjoying new foods/recipes |
|  |  | Limiting animal products / importance animal welfare |
|  | Grocery shopping style | Visiting multiple stores |
|  |  | Preferences for one store |
|  |  | Use of shopping list |
|  |  | No shopping list |
|  |  | Impulsive shopping |
|  |  | Non-impulsive shopping |
|  |  | Multiple times a week |
|  |  | Weekly groceries at once |
|  |  | Alternating at once/multiple times a week |
|  |  | Online grocery shopping |
|  |  | Avoiding temptations |
|  |  | Value in relationship with food retailer |
|  |  | Use of street food market(s) |
|  |  | Minimalizing food packing material |
|  |  | Reading nutrition labels |
|  |  | Stockpiling |
|  |  | Help with groceries |
|  | Food prices | Income (to) spent on food/willingness-to-pay |
|  |  | Influences of price promotions/advertisement |
|  |  | Getting food for free |
|  | Food preparation | Cooking skills |
|  |  | Frequency of Home cooking |
|  |  | Ordering food/ going out for meals |
|  |  | Minimally or not ordering food/going out for meals |
|  |  | Food prepping |
|  |  | Partner cooks |
|  | Importance of food | Making food a priority/ dedicating time |
|  |  | Positive attitude towards food and related activities |
|  |  | Negative attitude towards food and related activities |
|  |  | Functional |
|  |  | Comfort food |
|  |  | Pleasure |
|  |  | Absence of appetite/ picky eating |
|  |  | Social |
|  |  | Time-related barriers |
|  | Social influences | Social support |
|  |  | Shame/ social control |
|  |  | Social barriers |
|  |  | Self-efficacy |
|  |  | Media |
|  |  | Desire for social support/external motivation |
|  |  | Household eating rules |
|  |  | Preferences of others |
|  |  | Watching others eat |
| Eating Behaviors | Eating habits | Uncontrolled and emotional eating |
|  |  | Cognitive restraint of eating |
|  |  | Self-imposed rules/constraint |
|  |  | Giving into temptations |
|  |  | Based on satiety |
|  | Habit strength | Habit strength related to healthy food intake |
|  |  | Strong habit related to other foods |
|  |  | Consistent in dietary habits |
|  | Parental upbringing dietary habits | Traditional healthy upbringing |
|  |  | Exposure to unhealthy foods/less supportive environment |
|  |  | Cooking |
|  |  | Dieting as a child |
|  |  | Snack consumption |
|  |  | Beverage consumption |
|  |  | Experienced various cultures |
|  | Transitions dietary habits | Importance of health: prevention |
|  |  | Importance of health: to improve health |
|  |  | Importance of animal welfare |
|  |  | Education |
|  |  | After children |
|  |  | During (transition into) adulthood |
|  |  | After divorce/when in new relationship |
|  |  | Moving |
|  |  | Stopped work/sport routine |
|  |  | Due to medical reasons |
|  |  | Stopped smoking |
|  | Eating occasions | Frequency or timing of eating |
|  |  | Eating with others |
|  | Consumption of snacks | Own snack consumption |
|  |  | Minimal snack consumption |
|  |  | Children’s’ snack consumption |
|  |  | Compensation for snack eating |
|  | Dieting | Aiming to lose weight |
|  |  | Dieting in social environment |
|  |  | Avoiding sugar/ carbohydrates |
|  |  | (History of) eating disorder/ uncontrolled snacking behavior |
|  |  | Lifelong dieting |
|  |  | (Partly) plant based |
| Dietary Intake | Perceived dietary intake | Perceived healthiness of dietary intake |
|  |  | Perceived common dietary intake |
|  |  | Perceived variation in pattern |
|  | Dietary components | Vegetables |
|  |  | Fruits |
|  |  | Grains |
|  |  | Legumes |
|  |  | Nuts |
|  |  | Dairy |
|  |  | Fish |
|  |  | Tea |
|  |  | Coffee |
|  |  | Fats |
|  |  | Meat |
|  |  | Sugary beverages |
|  |  | Alcohol |
|  |  | Salt |
|  |  | Potato |
|  |  | Water |
|  |  | Sugar |
|  |  | Soup or bouillon |
|  |  | Egg |
|  |  | Meat substitutes |
